# Supplementary material for: The beetle amnion and serosa functionally interact as apposed epithelia
Source: eLife. 2016 Jan 29;5:e13834. doi: 10.7554/eLife.13834 (PMC4786423; doi:10.7554/eLife.13834)
Supplement: Supplementary file 1. — DOI: http://dx.doi.org/10.7554/eLife.13834.017 [file elife-13834-supp1.zip › Supplementry_File 1.pdf]

“The beetle amnion and serosa functionally interact as apposed epithelia”

Supplementary file 1.
Acquisition parameters for featured mDSLM light sheet experiments.

Dataset	Amnion x Heart	Serosa x Heart	Amnion	Serosa
Microscope	mDSLM (EMBLEM, Germany)			
Excitation objective	2.5x NA 0.06 EC Plan-Neofluar (Carl Zeiss, Germany)			
Detection objective	10x NA 0.3 W N-achroplan (Carl Zeiss, Germany)			
Illumination laser	488 nm diode laser, 20mW max. pulsed output power (PhoxX 488-20, Omicron GmbH, Germany)			
Emission filter	525/45 bandpass (525/45 Brightline HC Semrock, USA).			
Camera	1392x1040 CCD, pixel size 6.45 x 6.45μm (Andor Clara, Oxford Instruments, UK)			
GEKU line	Amnion (HC079) x Heart (G04609)	Serosa (G12424) x Heart (G04609)	Amnion (HC079) (homozygous)	Serosa (G12424) (homozygous)
Dataset unique identifier	TM12162330	TM26171836	TM03151712	TM08150418
Date acquired	01/12/2014	26/11/2014	03/06/2015	08/06/2015
Agarose (%)	1.4	1.0	1.4	1.4
Acquisition temperature (°C)	19.5 ± 1	19.5 ± 1	30.0 ± 0.1	30.0 ± 0.1
Laser power close to embryo (μW)	0.87	1.24	0.75	0.86
Exposure time (ms)	55	55	55	55
Z-spacing (μm)	2.58	2.58	2.58	2.58
Number of slices per time point	68	78	90	101
Z-Stack depth (μm)	175	200	230	260
Total number of time points	4320	4320	1304	1265
Time between time points (s)	20	20	120	120
Total duration of TL (hh:mm)	24:00	24:00	43:28	42:10
Presented in	Fig. 4G; Figure 4-figure supplement 1A; Movie S3	Figure 4-figure supplement 1B; Movie S3	Fig. 4D1-D2; Figure 2-figure supplement 1E1-E5	Fig. 4E1-E2; Figure 2-figure supplement 1D1-D5
Hatched?* (yes/no)	yes	yes	yes	yes

*After time lapse imaging, the agarose core containing the embryo was moved to 1x PBS at room temperature and inspected at daily intervals until hatching was confirmed.
